# A novel use of PICO dressing with sterile sponge for the treatment of a deep ulcerated wound in a breast cancer patient undergoing chemotherapy

**DOI:** 10.1093/jscr/rjad228

**Published:** 2023-04-28

**Authors:** Chien Lin Soh, Habib Tafazal

**Affiliations:** School of Clinical Medicine, University of Cambridge, Cambridge, UK; North West Anglia NHS Foundation Trust, Breast Unit, Peterborough City Hospital, Peterborough PE3 9GZ, UK

**Keywords:** Breast wound, Breast ulcer, Breast chemotherapy, Outpatient, Negative pressure wound treatment system

## Abstract

Wound management in patients with deep ulcerated wounds can be challenging, especially in the context of an outpatient setting. This is further confounded in patients undergoing chemotherapy. There is a lack of literature on the outpatient management of ulcerated breast wounds in patients having neo-adjuvant chemotherapy. This case report describes the use of a negative pressure wound treatment system leading to satisfactory wound healing and ultimately improving a patient’s quality-of-life during chemotherapy.

## INTRODUCTION

Wound management in patients with deep ulcerated wounds can be difficult for patients undergoing neo-adjuvant chemotherapy for the treatment of breast cancer, especially in the context of an outpatient setting. This case report demonstrates the use of a negative pressure wound treatment system to address such a challenge in an outpatient setting.

## CASE REPORT

**Figure 1:**
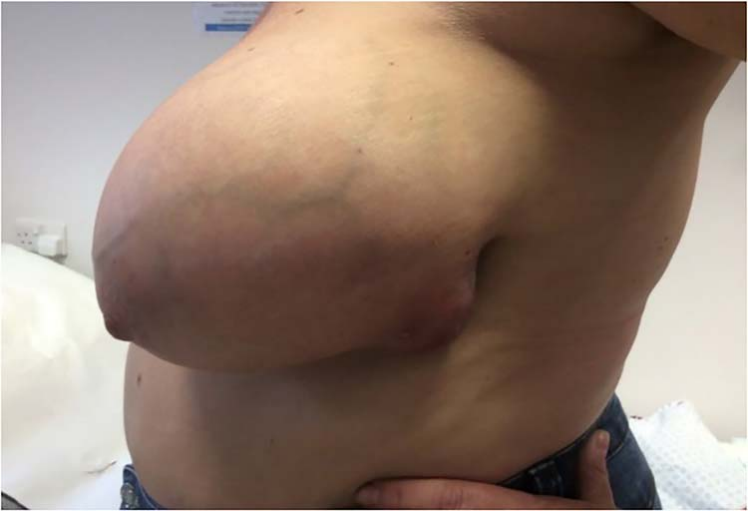
Left breast lump on first presentation.

A 47-year-old female patient presented to the breast unit with a 1-month history of a left-sided breast lump emerging to the skin (Fig. 1). The left breast wound was treated by the general practitioner previously with antibiotics with no response. She had no past medical or surgical history and was not on regular medication. She had a family history of cancer—her mother had breast cancer in her 50s and her maternal grandmother had ovarian cancer.

Upon examination and investigation by the breast team, the breast cancer was described as suspicious on mammogram. A further ultrasound demonstrated a 7 cm solid mass with abnormal axillary lymph nodes, which were all biopsied and shown to be benign. Histology of the tumour showed G3 invasive ductal carcinoma no-special-type, oestrogen receptor negative, progesterone receptor negative and Herceptin-2 (Her-2) receptor positive.

A staging computerized tomography (CT) scan was requested, and the patient was referred for neo-adjuvant therapy. The CT showed locally advanced breast cancer with potential pectoralis involvement alongside two small indeterminate lung nodules. A multidisciplinary team discussion recommended neo-adjuvant chemotherapy along with anti-Her-2 treatment.

**Figure 2:**
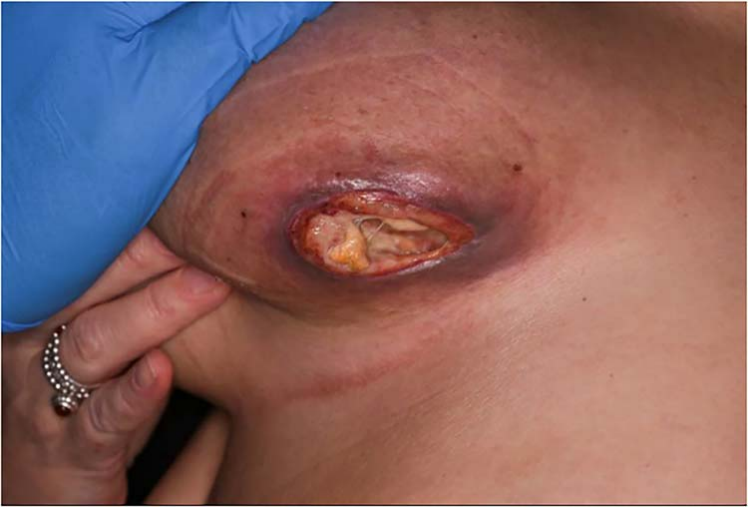
Left breast necrotic ulcer 1-week post-chemotherapy.

**Figure 3:**
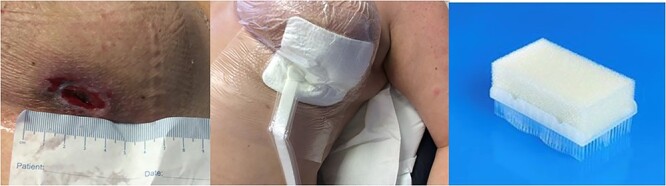
Sponge and PICO wound dressing in an outpatient breast clinic.

**Figure 4:**
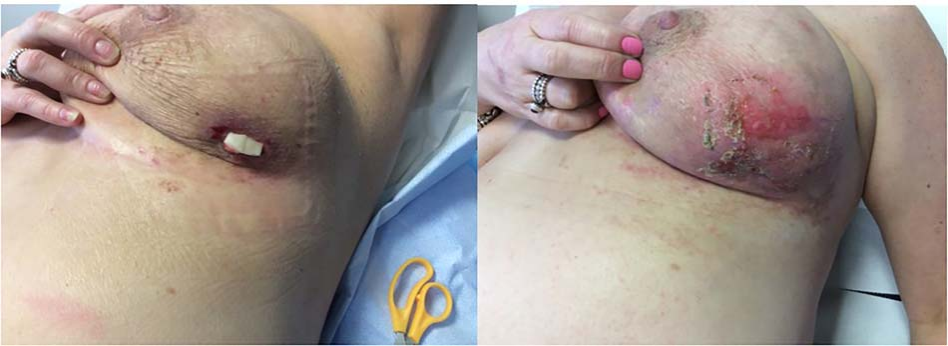
Outcome and healing of the breast wound.

The patient began chemotherapy and after one cycle the tumour responded very well to chemotherapy, however, the mass disappeared leaving a deep necrotic ulcer (Fig. 2). She attended the breast clinic where the necrotic ulcer was examined and treated with a sterile sponge from a scrub brush and PICO negative pressure wound therapy (NPWT) (Smith and Nephew Healthcare, Hull, UK) in an outpatient setting (Fig. 3). After 8 weeks of weekly dressing, the wound healed with resolution of the ulcer (Fig. 4). The patient described satisfaction with the clinical and cosmetic outcome of the treatment. The patient also avoided costly daily visits to the breast unit or surgical intervention through the use of this innovative technique.

The patient underwent mastectomy and sentinel lymph node biopsy without immediate reconstruction. On final histology it was noted that the patient’s tumour had a pathological complete response with two negative lymph nodes.

## DISCUSSION

This case report describes an elegant and cost-effective modification to the use of negative pressure wound therapy to address a deeply ulcerated breast wound in an outpatient setting. The challenges of wound management can be further compounded by the lack of consensus or national guidelines on post-chemotherapy breast ulcers. This unique case report adds to the body of literature on NPWT in deep breast ulcers, ultimately resulting in cost-savings, effectiveness and patient satisfaction.

The PICO negative pressure wound dressing systems have been advised by NICE technological guidance to treat closed surgical incisions in the NHS [1]. NPWT and PICO dressings provide continuous negative pressure on sealed wounds, absorbing exudate and acting as a physical barrier to allow discharge to evaporate [2]. Other negative pressure wound management systems absorb wound exudate into an attached cannister. PICO dressings in comparison absorb any exudate into the foam dressing itself. The use of NPWT allows reduction of admission to hospital, extra operative procedures and promotes better quality-of-life for the patients. The use of NPWT has been widely described in a variety of other surgical and medical specialities with significant benefits [2].

Adverse complications from breast cancer treatment such as neo-adjuvant chemotherapy is detrimental to the patient’s clinical outcome and quality-of-life [3]. Although generally efficacious and safe, some patients may experience wound healing complications [4]. Oncoplastic departments have described the lack of consensus for prophylactic negative pressure dressings in closed wounds, and there are no guidelines regarding the use of these systems to treat outpatient complications. [5] Quality-of-life metrics are important considerations for the patient and can influence the choice of technique. The patient ultimately avoided numerous trips to the breast unit for daily dressing change and had a completely healed wound whilst still receiving chemotherapy. The reduction in number of clinic visits was a significant benefit for the patient.

The PICO system has previously described in the literature in plastic surgery departments for post-operative patients [2] with demonstrated clinical and financial benefit with no dressing failure for all patients. Breast surgery departments internationally have also used the PICO system post-operatively to reduce complication frequency from common to rare complications [6–9]. Some departments describe stratifying risk of complications and adjusting use of the negative pressure dressings for higher risk operations [10]. This case report seeks to add evidence to the development of such guidelines for future management of complex breast ulcers. Further research on a larger scale must be completed to build the body of evidence supporting the use of NPWT in such applications.

This case report describes the successful use of the PICO dressing system in an outpatient setting to treat a deep necrotic ulcer with a satisfactory outcome. The patient ultimately avoided numerous trips to the breast unit for daily dressing change and had a completely healed wound whilst still receiving chemotherapy. This has led to a better quality-of-life in a setting where patients can become quite unwell with other complications of treatment. In addition to this, following her surgery the patient had a pathological complete response to treatment.

## Data Availability

There was no data processed as part of this case report.
